# Blood Types May Be Involved in the Occurrence of Coronary Heart Disease Through Serum Lipids

**DOI:** 10.1155/bmri/6113514

**Published:** 2026-03-20

**Authors:** Chen Jiang, JianHui Huang, Hui Cong

**Affiliations:** ^1^ Department of Blood Transfusion, Affiliated Hospital of Nantong University, Nantong, Jiangsu Province, China, ahnmc.com; ^2^ Department of Pathology, Affiliated Hospital of Nantong University, Nantong, Jiangsu Province, China, ahnmc.com; ^3^ Department of Blood Transfusion and Department of Laboratory Medicine, Affiliated Hospital of Nantong University, Nantong, Jiangsu Province, China, ahnmc.com

**Keywords:** ABO blood types, CHD, LDL-C, TC, TG

## Abstract

**Background:**

ABO blood types have important clinical significance in medicine. In this paper, our primary objective is to evaluate the association between ABO blood types and coronary heart disease (CHD) in the Chinese population. The secondary objective is to explore whether blood lipids may serve as a potential influencing factor in the relationship between blood types and CHD.

**Methods:**

We collected data from 5188 CHD patients to assess the distribution of blood types between CHD patients and blood donors, and performed binary logistic regression analysis to explore the correlation between blood types and CHD. Subgroup analyses and interactions were conducted to verify the robustness of the findings. Serum biological markers of CHD across different blood types were compared to explore potential underlying mechanisms.

**Results:**

The proportion of Type A was higher (*p* = 0.012), and Type O was lower (*p* = 0.047) in CHD. Logistic regression modeling analyses showed that Type A had a significantly increased risk of CHD by 24% compared with non‐A (OR [95% CI] 1.24: 1.05–1.45, *p* = 0.009). Blood Type A had a consistent effect on the risk of CHD across all subgroups, with no significant interaction observed (*p* for interaction > 0.1). Further, serum levels of total cholesterol (TC), triglyceride (TG), and low‐density lipoprotein cholesterol (LDL‐C) were found to be significantly higher in individuals with Type A CHD compared with those with non‐A blood types (*p* = 0.02, *p* = 0.045, *p* = 0.019).

**Conclusions:**

Type A is an independent risk factor for CHD, and CHD patients with Type A exhibit higher blood lipid levels, suggesting that blood type antigens may be involved in the occurrence of CHD through the lipid pathway.

## 1. Introduction

In 1901, Landsteiner defined the ABO blood types as the first recognized human blood type system. In addition to being expressed on erythrocytes, ABO blood type antigens are highly expressed on human cells and tissues. There has been an uninterrupted flow of research into the relevance of the blood types system to various diseases. The clinical value of blood types has extended beyond the traditional concepts of transfusion and transplantation medicine [1, 2]. Literature has demonstrated significant associations between blood types and various diseases, including tumors [3], infectious diseases [4], and thrombosis [5]. A comprehensive study showed that blood types were associated with heart failure (HF) and myocardial infarction (MI) [6]. Cardiovascular diseases are associated with high morbidity and mortality rates, and their continued rise in prevalence places a significant burden on national healthcare systems [7]. CHD is the leading cause of death worldwide, with lipids [8], hypertension and diabetes mellitus [9], cigarette smoking [9], and family history being the major risk factors [10]. Several studies have established a link between blood types and cardiovascular disease [11]. However, the study of its correlation with CHD remains somewhat controversial; therfore, we will delve into whether blood type is associated with CHD and preliminarily explore its possible mechanisms of association.

## 2. Materials and Methods

### 2.1. General Information and Study Design

A total of 5188 CHD patients, who attended the Affiliated Hospital of Nantong University and were clearly diagnosed by coronary angiography between January 2012 and December 2021, were collected and underwent retrospective investigation. Blood samples were collected after the patient′s admission and before undergoing coronary angiography and any major revascularization interventions. Inclusion criteria included patients aged > 18 years on first admission. Exclusion criteria included the presence of chronic inflammatory diseases (such as active infections and autoimmune diseases) and ABO blood type deficiency. Patients′ basic information, ABO blood types, and serum biomarker results were obtained from the hospital information system. This study is a retrospective design and did not systematically collect and adjust the use of all lipid‐lowering drugs (especially statins), antiplatelet drugs, and so on.

The population of blood donors at blood stations usually undergoes basic health screening (such as excluding active infections and severe chronic diseases) and can be considered a large, relatively healthy sample from the community, which is often used as a convenient and effective source of control in epidemiological studies. In view of this, we collected 255,748 cases of blood donors from Nantong Central Blood Station as a source of healthy population. Due to the large sample size of this population, we can obtain a representative subset based on demographic characteristics through simple random sampling to minimize selection bias when comparing with the case group. However, we fully recognize the inherent and systematic differences in age, gender, and underlying health status between them and CHD patients (e.g., blood donors are usually younger and healthier). To address this limitation, we have taken the following measures in our analysis: (1) using random sampling to construct a control sample, and (2) in all association analyses (logistic regression), age and gender—the two most critical demographic confounding factors—were forcibly adjusted. Despite these statistical adjustments, residual confounding caused by differences in unmeasured health‐related factors between the two groups may still exist, which observational studies based on such control designs cannot completely avoid. Due to the large gap between the sample sizes of blood donors and CHD, in order to eliminate the heterogeneity interference that could lead to the distortion of statistical tests, we selected 4557 individuals from the blood donors via simple random sampling to participate in the follow‐up study.

**Figure 1:**
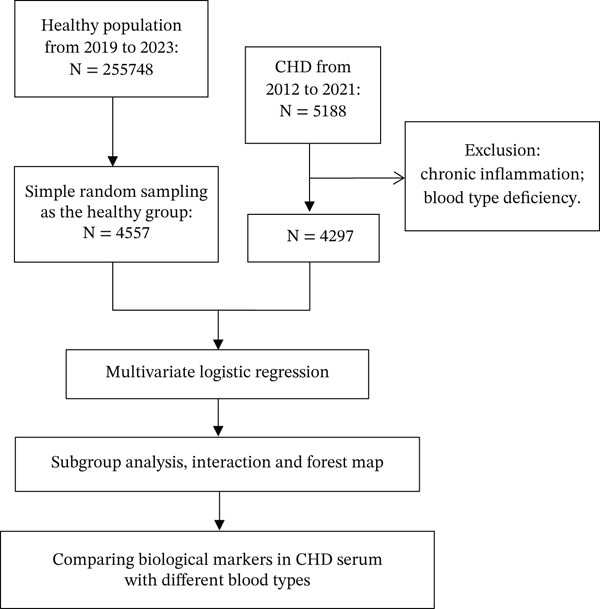
Flowchart detailing the selection process for patients included in this retrospective analysis.

We first assessed the demographic and blood types distribution characteristics of blood donors and CHD patients. We then performed binary logistic regression analyses to develop two models adjusted for age and gender to explore whether blood types were an independent risk factor for CHD. We performed subgroup analyses and interaction tests according to gender and age to validate the robustness of our findings and generated forest plots for presentation. The comparison of routine serum biological markers in CHD patients with different blood types is shown in Figure 1.

### 2.2. Instruments and Reagents

Subjects′ venous blood was collected according to the purpose and requirements of the test into test tubes containing separating gel and EDTA‐K_2_, respectively. Test tubes containing separating gel were centrifuged at 2068 g for 10 min within 2 h after blood coagulation, and the separated serum was used to test each biochemical index: lactate dehydrogenase (LDH, lactate‐substrate method), total bilirubin (T‐BIL, diazonium salt method), direct bilirubin (D‐BIL, diazonium salt method), total protein (TP, biuret method), albumin (ALB, bromocresol green method), TC (cholesterol oxidase method), triglyceride (TG, GPO‐POD method), LDL‐C (direct method), and high‐density lipoprotein cholesterol (HDL‐C, direct method). The detection instruments are used in the U.S. Beckman Coulter AU5800 fully automatic biochemical pipeline testing. The reagents were Beckman Coulter original kits and calibrators, and the quality control products were two levels of serum chemical quality control products from Bio‐Rad. A fully automated hemocytometer, Sysmex‐2100 (Sysmex, Japan), was used for whole‐cell counting. Microcolumn agglutination test was performed to identify the ABO blood types of the subjects (Soochow University Xaer Limited Liability Company). All the tests were performed under normal condition of the instruments and reagents and under in‐house quality control in strict accordance with the instrument and reagent standard operating procedures (SOPs). Original matching kits and calibrators were used for the tests. All necessary biosafety precautions and infection control protocols were followed during blood collection and testing.

The study was conducted in accordance with the Declaration of Helsinki and was approved by the Ethics Committee of Nantong University Hospital (2024‐K200‐01). Written informed consent was waived due to the retrospective nature of the study.

### 2.3. Statistical Analysis

Data are presented as mean ± standard deviation (SD) or median (interquartile range [IQR]) for continuous variables and as frequency or percentage for categorical variables. For baseline characteristics analysis, the statistical differences among blood types were tested with *t*‐test or one‐way ANOVA for continuous variables and chi‐square or fisher test for categorical variables. This study is an observational exploratory analysis, and no formal mediation effect test was performed. Statistical analyses were performed using R Version 3.3.2 (http://www.R-project.org, R Foundation). Statistical significance was defined as a two‐tailed *p* value < 0.05.

This study is an observational exploratory analysis aimed at describing associations rather than inferring causality. The discussion on “influence” in the article is based on statistical associations between variables and has not undergone formal mediation tests.

## 3. Results

### 3.1. Demographic Characteristics and Blood Types Distribution of the Healthy and Sample Populations

The results showed that the ABO blood types distribution among overall blood donors was O (31.5%) > A (30.8%) > B (27.9%) > AB (9.8%). The sampled population was not significantly different from the overall blood donors in terms of gender, age, and blood types distribution (all *p* > 0.2, as shown in Table 1). This indicates that the sample population effectively represents the overall blood donors and excludes the interference of sampling bias in the subsequent analysis.

**Table 1:** Demographic characteristics and blood type distribution of blood donors and sample population.

Variables	Sample healthy group (*n* = 4557)	Blood donors (*n* = 255,748)	*p*	Statistic
Gender, *n* (%)			0.233	1.423
Female	1974 (43.3)	113,049 (44.2)		
Male	2583 (56.7)	142,699 (55.8)		
Age, mean ± SD	38.0 ± 11.9	38.0 ± 11.9	0.987	0
Blood types, *n* (%)			0.394	2.982
A	1367 (30)	78,745 (30.8)		
B	1253 (27.5)	71,349 (27.9)		
O	1469 (32.2)	80,649 (31.5)		
AB	468 (10.3)	25,005 (9.8)		

### 3.2. Demographic and Blood Types Distribution Characteristics of Blood Donors and CHD Patients

Compared with the blood donors, the CHD population had an older mean age (65.9 ± 10.9 years vs. 38.0 ± 11.9) and a higher proportion of males (66.8% vs. 56.7%, all *p* < 0.001). Although we adjusted for age and gender through statistical models, there are inherent and significant differences in these demographic characteristics between the blood donor population and CHD patients. This difference may reflect deeper differences in health status and lifestyle, which cannot be fully controlled through simple statistical adjustments and may result in residual confounding. The distribution of the CHD blood types was characterized by: A (32.5%) > O (30.3%) > B (27.8%) > AB (9.5%), which was statistically different from the distribution of blood types in the blood donors (*p* = 0.038), which had the highest proportion of blood Type O. We dichotomized the blood types and found that the proportion of blood Type A was significantly higher in the CHD group than in the blood donors (32.5% vs. 30%, *p* = 0.012), and the proportion of blood Type O was significantly lower than in the blood donors (30.3% vs. 32.2%, *p* = 0.047), which was statistically different (as shown in Table 2).

**Table 2:** Demographic characteristics and blood type distribution of sample population and CHD.

Variables	Total (*n* = 8854)	Sample healthy group (*n* = 4557)	CHD (*n* = 4297)	*p*	Statistic
Gender, *n* (%)				< 0.001	95.08
Female	3402 (38.4)	1974 (43.3)	1428 (33.2)		
Male	5452 (61.6)	2583 (56.7)	2869 (66.8)		
Age, mean ± SD	51.6 ± 18.0	38.0 ± 11.9	65.9 ± 10.9	< 0.001	13,088.829

Blood types, n (%)				0.038	8.427
A	2762 (31.2)	1367 (30)	1395 (32.5)		
B	2446 (27.6)	1253 (27.5)	1193 (27.8)		
O	2770 (31.3)	1469 (32.2)	1301 (30.3)		
AB	876 (9.9)	468 (10.3)	408 (9.5)		

Blood types binary classification, *n* (%)					
Type non‐A	6092 (68.8)	3190 (70)	2902 (67.5)	0.012	6.27
Type A	2762 (31.2)	1367 (30)	1395 (32.5)		
Type non‐B	6408 (72.4)	3304 (72.5)	3104 (72.2)	0.779	0.079
Type B	2446 (27.6)	1253 (27.5)	1193 (27.8)		
Type non‐O	6084 (68.7)	3088 (67.8)	2996 (69.7)	0.047	3.949
Type O	2770 (31.3)	1469 (32.2)	1301 (30.3)		
Type non‐AB	7978 (90.1)	4089 (89.7)	3889 (90.5)	0.222	1.49
Type AB	876 (9.9)	468 (10.3)	408 (9.5)		

### 3.3. Logistic Regression Analysis of A or Non‐A and O or Non‐O Blood Types and CHD

Based on the results in Table 2, we developed logistic regression models to assess the independent effects of A versus non‐A and O versus non‐O blood types on CHD. When the two confounders of age and gender were not adjusted for, blood type A significantly increased the risk of CHD by 12% (OR [95% CI] 1.12: 1.03–1.23, *p* = 0.012) and blood Type O decreased the risk of CHD by 9% (OR [95% CI] 0.91: 0.83–0.99, *p* = 0.047) compared with non‐A. The results changed when further statistical adjustments were made by including two confounders, age and gender. Blood Type A had a significant 24% increased risk of CHD compared with non‐A (OR [95% CI] 1.24: 1.05–1.45, *p* = 0.009); and although the effect of blood Type O on CHD was no longer statistically significant, the risk of CHD decreased by 13% (OR [95% CI] 0.87: 0.74–1.02, *p* = 0.086). The results suggest that blood Type A is an independent risk factor for CHD and blood Type O shows a trend associated with lower risk of CHD, but this effect is affected by age and gender and is not sufficiently stable (as shown in Table 3).

**Table 3:** Logistic regression for Types A versus non‐A and O versus non*-*O in CHD.

Variables	*n*.total	*n*.event_%	Crude. OR (95% CI)	Crude. *p*	Adj. OR (95% CI)	Adj. *p*
Type non‐A	6092	2902 (47.6)	1(Ref)		1(Ref)	
Type A	2762	1395 (50.5)	1.12 (1.03–1.23)	0.012	1.24 (1.05–1.45)	0.009
Type non‐O	6084	2996 (49.2)	1(Ref)		1(Ref)	
Type O	2770	1301 (47)	0.91 (0.83–1)	0.047	0.87 (0.74–1.02)	0.086

*Note:* Crude models: unadjusted gender and age; Adj. models: adjusted gender and age.

### 3.4. Subgroup Analysis of the Association Between Blood Type A and CHD

**Figure 2:**
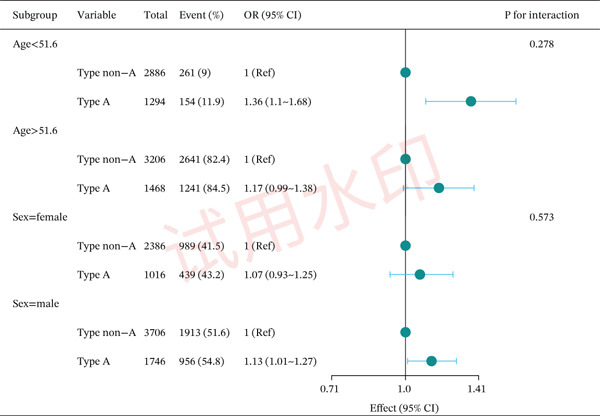
Subgroup multivariable analysis of the association between type A and CHD according to age and gender.

We further verified the stability of the effect of blood Type A on CHD. We dichotomized age using a mean age of 51.6 as the cutoff point, subgrouped by age and gender to assess the effect of A versus non‐A on CHD, and tested for interactions between subgroups using the likelihood ratio test. The results showed that blood Type A had a consistent effect on CHD risk across all subgroups, with no significant interaction observed (all *p* values for interaction > 0.2, as shown in Figure 2).

### 3.5. Routine Serum Biomarkers in CHD Patients With Different Blood Types

We sought to identify risk factors influencing the correlation between blood type and CHD. We compared the routine biological indicators of serum among patients with different blood types and found statistically significant differences in TC and LDL‐C in CHD patients between the four blood types (*p* = 0.012, *p* = 0.017, as shown in Table 4). Based on our previous study that blood Types A or non‐A and O or non‐O were correlated with CHD, we categorized the blood types into the A/non‐A and O/non‐O groups. Comparing these groups, we found that CHD patients with blood Type A had significantly higher levels of TC, TGs, and LDL‐C than those with non‐A blood types (*p* = 0.020, *p* = 0.045, *p* = 0.019), whereas CHD patients with blood Type O had lower levels than those with non‐O blood types, though these differences did not reach statistical significance (*p* = 0.451, *p* = 0.498, *p* = 0.192), as shown in Table 5.

**Table 4:** Routine serum biological markers of different blood types in CHD.

Variables	Total (*n* = 4297)	A (*n* = 1395)	B (*n* = 1193)	O (*n* = 1301)	AB (*n* = 408)	*p*	Statistic
Age, mean ± SD	65.89 ± 10.91	65.76 ± 11.45	65.81 ± 10.56	66.28 ± 10.73	65.35 ± 10.62	0.403	0.976
Gender, *n* (%)						0.090	6.495
Female	1428 (33.2)	439 (31.5)	390 (32.7)	467 (35.9)	132 (32.4)		
Male	2868 (66.8)	956 (68.5)	803 (67.3)	833 (64.1)	276 (67.6)		
WBC, mean ± SD	6.65 ± 2.27	6.68 ± 2.27	6.67 ± 2.33	6.58 ± 2.21	6.73 ± 2.30	0.587	0.643
NE, mean ± SD	4.48 ± 2.05	4.51 ± 2.03	4.51 ± 2.13	4.41 ± 1.97	4.57 ± 2.09	0.420	0.940
MO, mean ± SD	0.45 ± 0.21	0.44 ± 0.22	0.45 ± 0.21	0.45 ± 0.20	0.44 ± 0.20	0.846	0.271
LC, mean ± SD	1.57 ± 0.62	1.58 ± 0.62	1.57 ± 0.64	1.57 ± 0.61	1.57 ± 0.63	0.985	0.051
PLT, mean ± SD	194.53 ± 60.92	193.76 ± 61.70	196.35 ± 65.00	194.02 ± 57.02	193.39 ± 57.85	0.685	0.496
PDW, mean ± SD	13.92 ± 2.82	14.04 ± 2.88	13.85 ± 2.77	13.88 ± 2.80	13.83 ± 2.84	0.299	1.225
PLCR, mean ± SD	32.40 ± 9.54	32.83 ± 9.75	31.94 ± 9.52	32.34 ± 9.36	32.58 ± 9.46	0.174	1.656
TP, mean ± SD	67.39 ± 6.36	67.55 ± 6.39	67.35 ± 6.42	67.21 ± 6.26	67.53 ± 6.41	0.564	0.680
ALB, mean ± SD	40.12 ± 4.38	40.09 ± 4.33	40.18 ± 4.41	40.03 ± 4.36	40.32 ± 4.53	0.659	0.534
MPV, mean ± SD	10.88 ± 1.25	10.92 ± 1.29	10.82 ± 1.22	10.88 ± 1.25	10.88 ± 1.22	0.303	1.213
LDH, median (IQR)	194.00 (166.00, 237.00)	194.00 (166.00, 237.00)	196.00 (166.00, 241.00)	193.00 (165.00, 233.00)	196.00 (166.25, 239.00)	0.550	2.110
TBIL, mean ± SD	13.33 ± 7.38	13.54 ± 7.85	13.51 ± 7.10	12.87 ± 7.26	13.52 ± 6.88	0.073	2.324
UBIL, median (IQR)	3.15 (1.70, 4.80)	3.10 (1.60, 4.90)	3.20 (1.80, 4.80)	3.10 (1.60, 4.73)	3.20 (1.70, 4.80)	0.732	1.288
TC, mean ± SD	4.14 ± 1.13	4.20 ± 1.11	4.06 ± 1.07	4.12 ± 1.16	4.22 ± 1.22	0.012	3.637
TG, median (IQR)	1.36 (0.99, 1.91)	1.39 (1.02, 1.96)	1.32 (0.96, 1.86)	1.34 (0.97, 1.90)	1.42 (0.99, 1.96)	0.082	6.706
HDL‐C, mean ± SD	1.08 ± 0.28	1.07 ± 0.28	1.08 ± 0.26	1.09 ± 0.30	1.09 ± 0.28	0.468	0.847
LDL‐C, mean ± SD	2.48 ± 0.88	2.53 ± 0.87	2.43 ± 0.84	2.45 ± 0.90	2.54 ± 0.97	0.017	3.382

**Table 5:** Routine serum biological indexes of Types A versus non‐A and O versus non‐O.

Variables	Total (*n* = 4297)	Non‐A (*n* = 2901)	A (*n* = 1395)	*p*	Statistic	Non‐O (*n* = 2996)	O (*n* = 1301)	*p*	Statistic
Age, mean ± SD	65.89 ± 10.91	65.96 ± 10.64	65.76 ± 11.45	0.59	0.29	65.73 ± 10.99	66.28 ± 10.73	0.125	2.361
Gender, *n* (%)				0.088	2.919			0.014	6.046
Female	1428 (33.2)	989 (34.1)	439 (31.5)			961 (32.1)	467 (35.9)		
Male	2868 (66.8)	1912 (65.9)	956 (68.5)			2035 (67.9)	833 (64.1)		
WBC, mean ± SD	6.65 ± 2.27	6.64 ± 2.27	6.68 ± 2.27	0.598	0.278	6.68 ± 2.30	6.58 ± 2.21	0.189	1.722
NE, mean ± SD	4.48 ± 2.05	4.47 ± 2.05	4.51 ± 2.03	0.642	0.216	4.52 ± 2.08	4.41 ± 1.97	0.112	2.52
MO, mean ± SD	0.45 ± 0.21	0.45 ± 0.20	0.44 ± 0.22	0.51	0.435	0.44 ± 0.21	0.45 ± 0.20	0.422	0.644
LC, mean ± SD	1.57 ± 0.62	1.57 ± 0.63	1.58 ± 0.62	0.704	0.144	1.57 ± 0.63	1.57 ± 0.61	0.794	0.068
PLT, mean ± SD	194.53 ± 60.92	194.90 ± 60.55	193.76 ± 61.70	0.572	0.319	194.75 ± 62.55	194.02 ± 57.02	0.72	0.128
PDW, mean ± SD	13.92 ± 2.82	13.86 ± 2.79	14.04 ± 2.88	0.059	3.572	13.93 ± 2.83	13.88 ± 2.80	0.556	0.347
PLCR, mean ± SD	32.40 ± 9.54	32.21 ± 9.44	32.83 ± 9.75	0.065	3.408	32.43 ± 9.63	32.34 ± 9.36	0.777	0.08
TP, mean ± SD	67.39 ± 6.36	67.32 ± 6.35	67.55 ± 6.39	0.266	1.238	67.47 ± 6.40	67.21 ± 6.26	0.236	1.405
ALB, mean ± SD	40.12 ± 4.38	40.13 ± 4.41	40.09 ± 4.33	0.771	0.085	40.16 ± 4.39	40.03 ± 4.36	0.392	0.734
MPV, mean ± SD	10.88 ± 1.25	10.86 ± 1.23	10.92 ± 1.29	0.143	2.145	10.87 ± 1.25	10.88 ± 1.25	0.899	0.016
LDH, median (IQR)	194.00 (166.00, 237.00)	195.00 (166.00, 237.00)	194.00 (166.00, 237.00)	0.668	0.184	195.00 (166.00, 239.00)	193.00 (165.00, 233.00)	0.304	1.056
TBIL, mean ± SD	13.33 ± 7.38	13.22 ± 7.15	13.54 ± 7.85	0.192	1.706	13.53 ± 7.43	12.87 ± 7.26	0.008	6.96
UBIL, median (IQR)	3.15 (1.70, 4.80)	3.20 (1.70, 4.80)	3.10 (1.60, 4.90)	0.895	0.018	3.20 (1.70, 4.80)	3.10 (1.60, 4.73)	0.351	0.871
TC, mean ± SD	4.14 ± 1.13	4.11 ± 1.13	4.20 ± 1.11	0.020	5.39	4.15 ± 1.11	4.12 ± 1.16	0.451	0.568
TG, median (IQR)	1.36 (0.99, 1.91)	1.34 (0.97, 1.90)	1.39 (1.02, 1.96)	0.045	4.022	1.37 (1.00, 1.92)	1.34 (0.97, 1.90)	0.498	0.459
HDL‐C, mean ± SD	1.08 ± 0.28	1.08 ± 0.28	1.07 ± 0.28	0.314	1.014	1.08 ± 0.27	1.09 ± 0.30	0.161	1.969
LDL‐C, mean ± SD	2.48 ± 0.88	2.46 ± 0.88	2.53 ± 0.87	0.019	5.541	2.49 ± 0.87	2.45 ± 0.90	0.192	1.702

## 4. Discussion

The composition of ABO blood types antigens mainly consists of glycoproteins and glycolipid complexes. Their molecular structure includes a polypeptide backbone and anisotropic oligosaccharide chains, and the antigenic differences among different blood type systems arise from the diversity of glycosylation modifications. Blood type antigen phenotypes (such as the A and B antigens in the ABO system) are not simple linear structures but are instead formed by discontinuous amino acids folded in space to produce conformational epitopes with varying antigenicity. The specificity of these epitopes is determined by the stereochemical conformation of glycosyltransferases.

Due to the structural dynamics, genetic unpredictability, and paradoxical immune recognition of blood types antigens, the association between blood types and different diseases, including those affecting the cardiovascular system, has been investigated for decades. Although more studies have supported the relationship between blood types and cardiovascular diseases [12], the findings vary. For example, more researchers have found that blood Type A is an independent risk factor for CHD, and blood Type O has a lower risk of developing CHD [13–15]. Another large prospective cohort study found that non‐O blood types had a higher risk of CHD than O blood types [16]. This seems to hypothesize that blood Type A is an independent risk factor for CHD, whereas blood Type O is a protective factor. However, a study by Amirzadegan [17] in an Iranian population found no significant difference in the frequencies of blood types in CHD patients and controls. In Bangladeshi Asian Indian population, AB blood types reduced the risk of CHD, whereas Type O was more common in CHD [18]. In our study, the blood type distribution was O (31.5%) > A (30.8%) > B (27.9%) > AB (9.8%), which is consistent with the blood type distribution characteristics of the Chinese population [19]. We observed that the proportion of Type A blood in CHD patients was significantly higher than that in healthy individuals, whereas the proportion of Type O blood was significantly lower. The research results support that Type A blood is an independent risk factor for CHD, whereas Type O blood shows a protective trend. The controversial relationship between different blood types and CHD may be attributed to multiple confounding factors, such as diabetes, hypertension, and smoking. Another important factor is race and genetic factors, which may exert different influence on the relationship between blood type and coronary artery involvement across different racial groups. Additionally, socioeconomic conditions, environment, and lifestyle choices may also play a role in shaping the correlation between blood types and CHD.

Some recent GWASs and meta‐analyses support this potential role for ABO genotypes in modulating circulating levels of total and LDL cholesterol, as well as phytosterols, established causal risk factors for atherosclerotic heart diseases [20–22]. Current scholars almost recognize a causal relationship between CHD and lipids, with studies showing higher TC, LDL‐C, and non‐HDL‐C in non‐O blood types and confirming that about 10% of the effect of non‐O types on susceptibility to coronary artery disease is mediated through LDL‐C [23]. A meta‐analysis of 46 lipid‐based GWAS reported an association between ABO SNPs and serum cholesterol levels. Total cholesterol was increased by 2.3 mg/dL in heterozygote individuals for the ABO rs651007 SNP when compared with major allele homozygotes (P = 8.66 × 10^−21^), whereas LDL cholesterol was increased by 2.05 mg/dL in heterozygote individuals for the ABO rs649129 SNP when compared with major allele homozygotes (P = 7.85 × 10^−22^) [24]. A GWAS found that the ABO locus showed genome‐wide significance for association with phytosterol levels. Specifically, rs657152, which tightly tags the O1 allele, was found to be associated with decreased levels of circulating phytosterols, and individuals with the O allele had decreased campesterol concentrations when compared with the A and B alleles. Furthermore, additional analyses revealed that rs657152 was associated with reduced CAD risk (P = 4.0 × 10^−5^) when compared with alleles that were associated with increased phytosterols [20].

Based on our research findings, we speculate that blood type may be related to serum lipid levels, which in turn affect the occurrence of CHD through blood lipids. However, this discovery must be interpreted with caution. First, this study is a cross‐sectional observational study, and due to limited data, it cannot establish a causal temporal or mediating relationship between blood lipids and blood type. Secondly, in addition to the lipid pathway, ABO blood type may also affect the occurrence of CHD through other mechanisms. It has been claimed that serum levels of Factor VIII‐VWF complex were 25% higher in individuals of the non‐O group than those of the O group. VWF plays an important role in hemostasis and thrombosis by mediating platelet adhesion to the vascular wall [25]. VWF is involved in platelet aggregation along with fibrinogen and plays a role in the development of atherosclerosis [26, 27]. This could explain why blood Type O exhibits a protective tendency compared with other blood types, but it does not account for why only Type A is a risk factor among non‐O blood types, whereas Types B and AB are not. We speculate that a possible reason for this phenomenon is that Type A antigens may enhance platelet aggregation and inflammatory responses that promote atherosclerotic plaque destabilization. Due to the rarity of AB type in the sample (only 9.5% in this study), this may limit statistical power to reach significance, even if there are biological risks. Another important explanation is genetic linkage imbalance. Scholars have found that ATP binding cassette 2 (ABCA2) plays a certain role in maintaining cholesterol balance and lipid homeostasis. Coincidentally, the ABCA2 gene locus and ABO blood type are both located on chromosome 9 at 9q34 [28, 29]. In the process of inheritance, these gene loci with similar positions may tend to be inherited as a whole to offspring, rather than being separated independently. Therefore, the association observed between ABO blood type and specific lipid phenotypes may not be directly caused by the ABO gene itself, but rather reflects its co‐inheritance with nearby functional genes. In other words, it is the “neighbors of blood type genes” at work. A study on the plasma proteome found a causal relationship between ABO antigens and CAD [30]. Evidence on whether these blood characteristics contribute to the association between blood types and CHD remains scarce. These findings provide a theoretical basis for the link between blood types and CHD. Therefore, although this study supports the association between blood type and CHD and specific lipid profiles, there is a complex network involving multiple factors and mechanisms behind it. The potential mechanism between blood types and CHD has not yet been adequately studied and fully confirmed.

Our research shows that the serum lipid levels of Type A CHD patients are significantly higher than those of non‐A CHD patients, wheras the serum lipid levels of Type O CHD patients are lower than those of non‐O. The logistic regression model also found that the risk of coronary heart disease in Type A and non‐A CHD patients is 24% higher, and the risk of CHD in Type O CHD patients is 13% lower than that in non‐O CHD patients. The “A antigen” of blood Type A and the “A antigen” of blood Type AB are not absolutely equivalent concepts in molecular serology. The majority of individuals with blood Type AB (A₁B) exhibit significantly lower A antigen density compared with those with blood Type A (A₁), and some AB individuals (A₂B) even lack the critical A₁ antigenic determinants. Therefore, in this study, the A antigen is considered a risk factor, whereas the association with blood Type AB is weakened or even absent, which is entirely consistent with the principles of immunogenetics. This provides clues for the hypothesis that dyslipidemia may be a potential contributing factor in the association between ABO blood type and CHD. In clinical practice, identifying individuals with additional, independent risk factors can help strengthen the management of traditional modifiable risk factors such as hyperlipidemia and hypertension. For example, for an individual with Type A blood with a critical high cholesterol level, the results of this study may provide additional considerations for clinicians to more actively recommend lifestyle interventions or closer monitoring of blood lipids. In clinical management, providing stronger and stricter lipid‐lowering treatment targets for such patients may bring greater clinical benefits. Although this strategy needs to be validated in prospective intervention studies, it provides a potential and worth exploring new perspective for individualized cardiovascular disease risk management based on blood type.

Finally, we should emphasize the limitations of our research. Firstly, there may be selection bias in the use of retrospective designs and blood donor controls, with limited comparability in terms of age and gender. Secondly, the most important limitation lies in the limited data. We only adjusted for age and gender, and a large number of known key clinical risk factors (such as hypertension, diabetes, smoking history, and BMI) and drug use information were not completely collected, including the use of lipid‐lowering drugs (especially statins) and antiplatelet drugs, which will directly affect blood lipid levels and cardiovascular risks. Therefore, the research results may be significantly affected by residual confounding; meanwhile, due to the lack of blood lipid data from blood donors, we are unable to conduct formal mediation analysis.

## 5. Conclusion

This study suggests that blood type may be independently associated with a higher risk of CHD in the Chinese population, while Type O may have a protective trend. In addition, CHD with Type A exhibits higher levels of TC, TG, and LDL‐C, providing preliminary support for the hypothesis that ABO blood types may partially participate in CHD development by affecting lipid metabolism. In the future, prospective studies should be conducted and more comprehensive confounding factors should be included to explore the association and potential mechanisms.

## Author Contributions

Chen Jiang conceptualized and designed the study, conducted data analysis, interpreted, and wrote the manuscript. JianHui Huang collected and assembled the data. Hui Cong guided the revision of the paper.

## Funding

This study was supported by InTec PRODUCTS, INC, (JSYK2024023)

## Disclosure

All authors read and approved the final version of the manuscript.

## Conflicts of Interest

The authors declare no conflicts of interest.

## Data Availability

The data presented in this study are available upon request from the corresponding author.
